# Revisiting COVID-19 policies: 10 evidence-based recommendations for where to go from here

**DOI:** 10.1186/s12889-021-12082-z

**Published:** 2021-11-13

**Authors:** Daniel T. Halperin, Norman Hearst, Stephen Hodgins, Robert C. Bailey, Jeffrey D. Klausner, Helen Jackson, Richard G. Wamai, Joseph A. Ladapo, Mead Over, Stefan Baral, Kevin Escandón, Monica Gandhi

**Affiliations:** 1 Gillings School of Global Public Health, University of North Carolina, Chapel Hill, NC USA; 2 Department of Family and Community Medicine, School of Medicine, University of California, San Francisco, CA USA; 3 School of Public Health, University of Alberta, Edmonton, AB Canada; 4 School of Public Health, University of Illinois, Chicago, IL USA; 5 Keck School of Medicine, University of Southern California, Los Angeles, CA USA; 6 Independent Consultant, Harare, Zimbabwe; 7 Integrated Initiative for Global Health, Northeastern University, Boston, MA USA; 8 School of Public Health, University of Nairobi, Nairobi, Kenya; 9 Division of General Internal Medicine and Health Services Research, David Geffen School of Medicine, University of California, Los Angeles, CA USA; 10 Center for Global Development, Washington, D.C, USA; 11 Department of Epidemiology, Johns Hopkins School of Public Health, Baltimore, MD USA; 12 School of Medicine, Universidad del Valle, Cali, Colombia; 13 Department of Microbiology, Universidad del Valle, Grupo de Investigación en Virus Emergentes VIREM, Cali, Colombia; 14 Division of HIV, Infectious Diseases, and Global Medicine, Department of Medicine, University of California, San Francisco, CA USA

**Keywords:** COVID-19, SARS-CoV-2, Public health, Vaccines, Harm reduction, Policy, Outdoor transmission, School closure, Pandemic, Pandemic preparedness, Evidence-based recommendations

## Abstract

**Background:**

Strategies to control coronavirus 2019 disease (COVID-19) have often been based on preliminary and limited data and have tended to be slow to evolve as new evidence emerges. Yet knowledge about COVID-19 has grown exponentially, and the expanding rollout of vaccines presents further opportunity to reassess the response to the pandemic more broadly.

**Main text:**

We review the latest evidence concerning 10 key COVID-19 policy and strategic areas, specifically addressing: 1) the expansion of equitable vaccine distribution, 2) the need to ease restrictions as hospitalization and mortality rates eventually fall, 3) the advantages of emphasizing educational and harm reduction approaches over coercive and punitive measures, 4) the need to encourage outdoor activities, 5) the imperative to reopen schools, 6) the far-reaching and long-term economic and psychosocial consequences of sustained lockdowns, 7) the excessive focus on surface disinfection and other ineffective measures, 8) the importance of reassessing testing policies and practices, 9) the need for increasing access to outpatient therapies and prophylactics, and 10) the necessity to better prepare for future pandemics.

**Conclusions:**

While remarkably effective vaccines have engendered great hope, some widely held assumptions underlying current policy approaches call for an evidence-based reassessment. COVID-19 will require ongoing mitigation for the foreseeable future as it transforms from a pandemic into an endemic infection, but maintaining a constant state of emergency is not viable. A more realistic public health approach is to adjust current mitigation goals to be more data-driven and to minimize unintended harms associated with unfocused or ineffective control efforts. Based on the latest evidence, we therefore present recommendations for refining 10 key policy areas, and for applying lessons learned from COVID-19 to prevent and prepare for future pandemics.

## Background

**Fig. 1:**
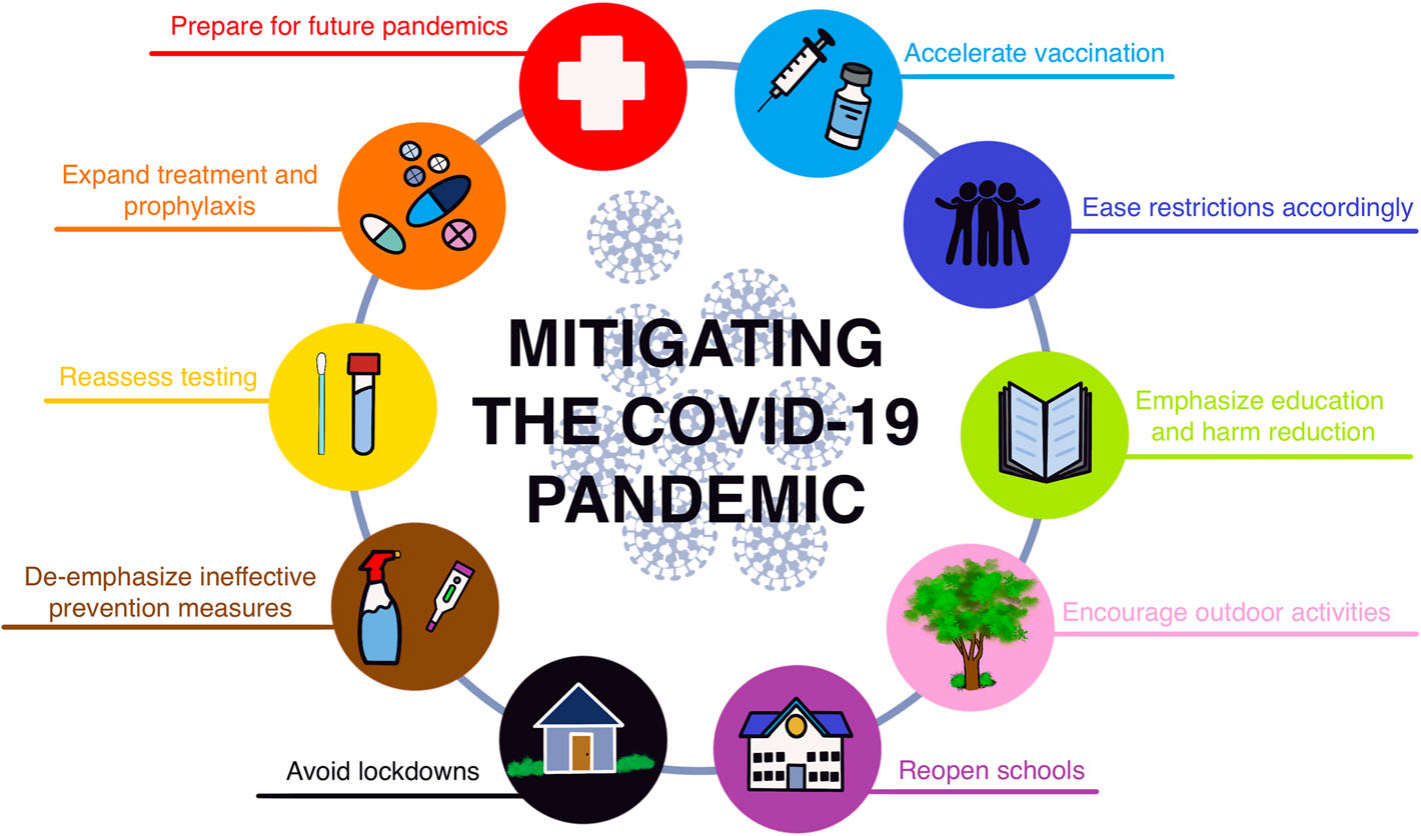
Evidence-based recommendations for 10 key COVID-19 policy and strategic areas. Figure designed by Karina Escandón

The coronavirus disease 2019 (COVID-19) pandemic has caused devastating loss of life and disrupted healthcare systems and daily life globally. By late October 2021, over 245 million confirmed severe acute respiratory syndrome coronavirus 2 (SARS-CoV-2) infection cases and over 4.9 million related deaths had been reported globally [1]. As the international vaccination rollout continues to expand [2], we call for a reexamination of existing mitigation approaches to adapt to emerging evidence on effectiveness and to minimize unintended consequences. COVID-19 vaccines have proven to be highly effective at preventing severe disease and mortality and, to a lesser extent, milder symptomatic and asymptomatic cases. While vaccination has ushered in great hope, the time is ripe to revisit the assumptions underlying some current interventions and to implement more context-sensitive, evidence-based policies. Accordingly, we review the available evidence regarding 10 key policy areas for which we recommend modification or refinement (Fig. 1).

One limitation of this review is the paucity of data from randomized controlled trials (RCTs) to measure the efficacy and effectiveness of COVID-19 prevention interventions. Aside from vaccines and therapeutics [3, 4], the only exceptions to date are two RCTs of masks [5, 6]. A trial conducted in Denmark found no statistically significant difference in infection rates between the group provided with and urged to wear surgical masks and a control group [5]. Meanwhile, a cluster RCT in Bangladesh found a statistically significant 9% reduction in symptomatic seroprevalence in villages where surgical masks were provided and their use promoted [6]. In this study, no significant decrease in symptomatic seroprevalence was observed in villages where cloth masks were promoted. Moreover, some public health interventions can be difficult or even impossible to definitely study with RCTs [7–10]. We therefore rely mainly on the best available observational data, despite limitations and potential biases, to suggest refinements to current approaches and policies.

### 1: Accelerate vaccination rollout

Even with the continuing emergence of viral variants, widespread vaccination remains the quickest and most powerful way to reduce the toll from COVID-19 and continue returning toward a greater sense of normality. Maximizing global vaccine production and equitable distribution must be the highest priority, with innovative mechanisms of financing and licensing production as required. The wealthier countries should largely pay for this ongoing effort as a humanitarian imperative as well as from enlightened self-interest. This could be modeled on the experience with AIDS, in which antiretroviral drugs are provided to poorer countries by bilateral and multilateral donors at discounted prices and/or through low-cost international generic production via waived patents [11, 12], while pharmaceutical companies continue to benefit financially in higher-income countries. Many countries have recently made encouraging promises in this regard, including at the June 2021 G7 Summit [13], but such promises will need to be kept if not exceeded.

Since vaccine supplies are still not adequate to meet the global population’s needs, they must be used as strategically and efficiently as possible. Such strategies include prioritizing vulnerable populations and healthcare workers (HCWs), and delaying doses for those with previous COVID-19 until those without prior immunity are vaccinated. Delaying the second dose of 2-dose vaccines for longer than the interval used in clinical trials may increase overall public health benefit by maximizing coverage with first doses more quickly and may also lead to greater immunogenicity [14–19]. The US Centers for Disease Control and Prevention (CDC) recommended that the second dose can be given up to 6 weeks following the first one [20], but implementing an even longer duration between doses, when necessary, is consistent with a population-health perspective. Several countries, such as Canada, have taken this approach of extending the duration between doses. Moreover, persons known to have been previously infected may defer vaccination for 6 months or perhaps even longer post-infection [21, 22], and when they get vaccinated, appear to require only 1 dose of a 2-dose vaccine regimen [23–26].

Such approaches will require careful implementation and messaging to minimize the potential risk of persons not getting vaccinated in the unconfirmed belief that they have already been infected, or not returning for a second dose in the mistaken belief that they are fully protected by a single dose. Other challenges facing some countries involve choosing between rapid application of less effective vaccines or waiting for the availability of better ones. Generally, those approaches that offer the most people some protection as quickly as possible should be followed. Furthermore, higher-income countries should refrain from distributing booster shots more broadly or frequently than necessary (e.g., for the immunocompetent general population), as this appears currently unjustified both scientifically and ethically [27, 28]. In late October 2021, it was estimated that globally about three times as many booster shots were being given per day compared to the total number of vaccine shots administered daily in lower-income countries [29].

Vaccination and other mitigation efforts must focus on protecting the most vulnerable through prioritizing the elderly, HCWs, and other essential workers. Additional criteria for determining which persons should be prioritized due to existing medical conditions must be evidence-based. Cardiometabolic comorbidities such as diabetes, chronic obstructive pulmonary disease, hypertension, and obesity are known risk factors strongly associated with increased COVID-19 severity and mortality [30–35]. But asthma, for example, turns out not to be a risk factor (and is probably even partially protective against death and other serious COVID-19 outcomes) [36–41]. In certain situations and particularly among groups at the highest risk of disease or exposure, vaccination mandates can be considered, e.g., for working in hospitals, nursing homes, prisons, or other high-risk settings [42, 43]. While we applaud the US and other countries for having joined the World Health Organization (WHO) COVAX Initiative, we urge high-income countries to also unilaterally deploy their soon-to-expire as well as other doses overseas and to join the WHO COVID-19 Technology Access Pool, which would allow other countries to produce patented vaccines, thereby expanding their availability in low and middle-income countries [11]. ﻿International governance of vaccine distribution is essential to address vaccine inequity and to maximize outcomes globally.

### 2: Gradually ease restrictions as vaccination expands

Accumulating real-world evidence is documenting the large extent to which COVID-19 vaccines reduce severe disease, hospitalizations, and mortality. Although asymptomatic infection and symptomatic disease were both greatly reduced by the vaccines in the context of the Alpha variant and earlier D614G mutants [18, 19, 44–53], more recent data during the ascendency of the Delta variant indicate reduced effectiveness against asymptomatic or mild infections [54–60]. However, the vaccine-induced protection against severe disease from the Delta variant appears to be remarkably intact across multiple settings, at over 90% [55, 59]. Declines in antibodies are expected over time following vaccination, but cellular memory (which enhances antibody production and protects against severe disease) appears to be much more durable [61, 62].

Once vaccination has been made widely and equitably available and rates of hospitalization and mortality eventually fall, it becomes untenable to expect the vaccinated to follow all current restrictions imposed mainly to protect those who decline vaccination. The same can be said regarding immunity following infection. Given the rarity of reinfection [7, 22, 63] and the duration of immunity post-infection (at least 6–12 months ) [21, 63–68], those with evidence of prior infection appear to be as immune as those who have been vaccinated [69–72].

Mass vaccination will accelerate achieving much greater pandemic control, allowing measures such as masking and physical distancing to be gradually relaxed [7]. It is critical to acknowledge the physical, psychological, sociopolitical, and other costs of enforcing restrictions and to begin easing them as hospitalization and death rates fall substantially, while remaining vigilant and ready to revisit such decisions if circumstances change significantly.

### 3: ﻿Emphasize education and harm reduction approaches over coercive and punitive measures

“Abstinence-only” approaches have not worked for AIDS or teen pregnancy prevention [73], nor have absolutist approaches worked well for preventing SARS-CoV-2 [74, 75]. Instead, prevention measures should be founded on the provision of accurate information, sensitively communicated, and informed by harm reduction approaches that are more effective and sustainable in the longer term [7, 74–76]. Harm reduction involves informing people how to assess and mitigate risk, while acknowledging the real-world conditions that may lead some persons to take calculated risks. One example of a successful mitigation campaign (prior to vaccines) is that of Japan’s 3 Cs, which generally did not shut down society, but instead advised the public to avoid *c*lose, sustained interactions in *c*rowded en*c*losed spaces [77]. Importantly, educating and motivating the public to adopt effective precautions, including vaccination, as opposed to coercive or punitive measures (e.g., shaming, fines or imprisonment, and even police violence) will be more effective and will help alleviate pandemic response fatigue [7, 78–80]. Accordingly, any restrictions and mandates, including vaccinations passports [81–83], should focus on high-risk situations and consider a number of scientific and ethical questions. Most importantly, COVID-19 measures should be formulated and reassessed based on the latest information, levels of ongoing threat, and resource availability. As mentioned above, vaccine mandates should be carefully focused and should take into account prior SARS-CoV-2 infection [22].

### 4: ﻿Encourage outdoor activities

Current evidence on SARS-CoV-2 transmission dynamics must inform policy recommendations for mitigation strategies and restrictions [84]. Unfortunately, lower-risk activities, especially those conducted in outdoor environments (e.g., parks, beaches, hiking trails, playgrounds), have often been discouraged or even prohibited [85–90]. The risk of SARS-CoV-2 transmission outdoors is vastly lower than indoors, with most studies finding the proportion of new cases attributable to outdoor exposure to be < 1% [7, 42, 91–94]. Policies should reflect this enormous difference in risk, including allowing access to outdoor spaces even during periods of severe restrictions and reserving mask mandates for indoor (and very crowded outdoor) situations [7], as recommended by the WHO and CDC [95–97]. Strongly encouraging outdoor activities and including nuance in public health recommendations (such as discouraging outdoor gatherings from leading to crowded indoor situations) is more consistent with the previously discussed harm reduction-based approaches [7, 98]. When weather or other factors preclude holding activities outdoors, windows should be kept open whenever possible, including in shared vehicles [99], and air ventilation (at least 4 air exchanges per hour) should be ensured to reduce the risk of transmission [100–102].

### 5: ﻿Reopen schools now

COVID-19 has caused by far the largest disruption to learning in recent history [103]. As the pandemic has unfolded, there is mounting evidence that the harm of keeping schools closed dwarfs any public health benefits [41, 104, 105]. By early 2020, most kindergarten-to-grade 12 (K-12) schools worldwide had closed for in-person instruction, and many remain shuttered over a year later [104, 106–109]. As of September 2021, based on United Nations Educational, Scientific and Cultural Organization (UNESCO) data [109], over 100 million students remained affected and 18 countries still had nationwide closures. There is no good substitute for in-person schooling [108]. Remote learning further exacerbates inequities, especially among communities with low resources, not only related to education but also to safety, wellbeing, social support, and nutrition [105, 108, 110–112].

Schools have not been shown to be major drivers of SARS-CoV-2 transmission, when studied in a variety of settings employing a range of mitigation strategies and intensity [106, 107, 113, 114]. However, their prolonged closure have had disastrous academic, psychosocial, and other harmful consequences on children, including access to essential services, especially in lower-income populations [41, 111, 115, 116]. Furthermore, contact tracing studies worldwide have found children are less likely to infect adults or other children, and that most SARS-CoV-2 infections among children are mild and are contracted at home or in the community, not at school [106, 107, 117–119].

In the US state of North Carolina prior to vaccine availability, 11 school districts (many in regions with high SARS-CoV-2 incidence) implemented in-person instruction accompanied by mitigation plans, for > 90,000 children over 9 weeks [117]. Across the 11 school districts, there were 773 community-acquired SARS-CoV-2 infections documented by reverse transcriptase-polymerase chain reaction (RT-PCR) testing, of which only 32 were identified as secondary cases, with no cases of within-school transmission from children to teachers or other adults. Among 17 US schools in rural Wisconsin also conducting in-person learning, with a range of precautions, SARS-CoV-2 incidence among students, teachers, and other staff members was lower than in the surrounding communities overall [118]. During 13 weeks in late 2020, 191 cases were identified among students and staff, of which only 7 (3.7%) cases (all among students) were traced to in-school transmission. In Sweden, where schools generally remained open (and masks have not been required) [120, 121], deaths of children aged 1–16 years were statistically similar in the 4 months before versus after COVID-19 arrived, and intensive care unit admission rates for teachers were comparable to those for other occupations [122]. Many other investigations, such as one among children aged 0 to 19 years in childcare facilities and schools in Baden-Württemberg, Germany, after the reopening of schools in May 2020, have also suggested that child-to-child transmission in school settings is uncommon [123]. To the extent that in-school transmission is an issue, especially given the continuing emergence of highly transmissible variants (e.g., Delta), vaccinating school staff is likely the most effective way to protect those at risk [124–126].

Also, after reviewing data indicating that 3 ft of physical distancing is sufficient [127], in March 2021 the CDC modified their guidelines accordingly, at least for elementary school settings [128]. A large-scale CDC study, comparing schools that mandated various interventions in late 2020 with ones that did not, found that while improving ventilation and requiring teachers and staff members to wear masks was associated with reduced SARS-CoV-2 incidence in schools, mandating students to wear masks was not [129]. Masking guidelines for children from major public health organizations differ, which has generated confusion. For instance, the CDC currently recommends that all children over age 2 wear masks indoors, while the WHO mask guidance applies to children over age 5, with a caveat that benefits from mask mandates at school may not outweigh the potential academic and psychosocial harms [130]. Despite the inconsistent data and guidelines, student masking in communities where rates of hospitalization and death remain high may be useful [113], if for no other reason than to help maintain the necessary consensus to keep schools open.

The emergence of variants does not warrant closing or delaying the reopening of schools unless compelling evidence unexpectedly indicates that a new mutation affects children in some substantially new way [131]. Reassuring data from high schools [106, 107, 117, 118, 122, 123] suggest that in-person classes also can be safely conducted in colleges, especially if combined with interventions to prevent outside-the-classroom transmission. As endorsed by the United Nations Children’s Fund (UNICEF) [132], no effort should be spared to keep students in classes, and closing schools should be a measure of last resort.

### 6: ﻿Avoid lockdowns

The cumulative evidence suggests that “sledge-hammer” lockdown approaches, such as the closing of all non-essential workplaces and schools, should be avoided in favor of more effective, carefully targeted “scalpel” public health strategies [7, 78, 133, 134]. Indiscriminate lockdowns have had far-reaching unintended consequences, disproportionately affecting socioeconomically disadvantaged and vulnerable populations. Other consequences include alarming increases in mental health problems (e.g., depression, anxiety, and social isolation), drug overdose, domestic violence, child abuse, weight gain, abuse by law enforcement in some places, and discontinuation of non-COVID-19 clinical services and prevention programs [41, 78, 110, 115, 134–139]. While substantial evidence highlights the deleterious impact of sustained lockdowns, the direct impact of SARS-CoV-2 transmission on disease outcomes, healthcare systems, and employment, particularly in the context of huge inequity, can also produce many of the same negative effects, even in the absence of official lockdowns [140, 141].

Tailored, context-sensitive interventions involving fewer economic, societal, and quality-of-life costs than lockdowns are likely more effective and minimize harm [7]. Non-pharmaceutical interventions such as physical distancing, improved ventilation, and effective indoor mask wearing are also more sustainable than broad stay-at-home orders [142–146]. Although emerging genetic SARS-CoV-2 variants may pose additional challenges [147], the biological and epidemiological evidence suggests that the same interventions will work to reduce their transmission. When lockdowns, isolation, or quarantine measures are mandated, economic hardship should be considered and paid sick/quarantine leaves and other types of support must be provided to affected workers, especially those who are most economically vulnerable [7].

### 7: ﻿De-emphasize excessive surface disinfection and other ineffective measures

The evidence is consistent that indirect contact (fomite) transmission is not a significant driver of SARS-CoV-2 spread [148–151], as acknowledged by the CDC [152]. Many routine disinfection rituals, including the ubiquitous usage of alcohol-based hand sanitizers and the excessive use of strong cleaning products, are unnecessary [41, 153]. Misuse of sanitizers, cleansers, and disinfectants has resulted in toxic reactions occasionally leading to hospitalization and even death [154–156]. Such hazardous disinfection practices include washing food products with bleach, applying household cleaning or disinfectant products to bare skin, mixing bleach solutions with vinegar or ammonia, and intentionally or accidentally inhaling or ingesting such products [155, 156]. Beyond being ineffective and occasionally dangerous, excessive cleaning rituals divert important resources, time, and energy from much more useful forms of prevention [151, 153]. There are also growing concerns about the potential longer-term impact on what many scientists have warned is the looming “next pandemic,” that of antimicrobial resistance [157, 158]. Similarly to the misplaced focus on disinfection rituals, public health authorities and the media must do a much better job of educating the public how the coronavirus is—and is not—typically transmitted [159, 160]. For example, fleeting encounters pose minimal risk, even from more transmissible variants^41^, ^78^.

Another pervasive practice, temperature screening—especially when using non-contact handheld cutaneous infrared thermometers—is often inaccurate due to environmental factors (e.g., subject-to-sensor distance, ambient temperature, humidity), operator-dependent performance, device variability, and feature changes in target subjects [161–165]. Furthermore, fever is a poor differentiator of the presence or absence of SARS-CoV-2 infection (and the use of antipyretic drugs may mask fever). The ubiquitous use of thermometers for permitting entry to public establishments is thus ineffective. A systematic review of studies regarding exit and entry screening practices (e.g., symptom questionnaires, body temperature measurement) during previous epidemics of influenza A(H1N1), Ebola, and severe acute respiratory syndrome (SARS) found extremely low or no utility in differentiating infected from uninfected [166]. For COVID-19, similar findings have been reported, with only a very small proportion of SARS-CoV-2 infection cases detected during such screening practices [167]. Again, such measures divert resources and attention away from much more effective strategies to control infection.

Furthermore, travel-related restrictions have clearly had a considerable impact on global trade and economies as well as on other systems, including those for international humanitarian responses [145]. Other negative consequences include generating a false sense of security, discouraging travelers from engaging transparently with authorities, and potentially disincentivizing open disclosure by countries during future outbreaks [131, 168]. Although a few countries (e.g., New Zealand, Australia, Taiwan, China), mainly island nations, have attempted SARS-CoV-2 elimination through use of robust quarantine and contact tracing measures [7, 131, 169], it makes little sense, from either an epidemiological or human rights perspective, to shut international land borders or require a negative RT-PCR test result for entry into countries where SARS-CoV-2 is already circulating widely. Similarly, the routine use of quarantine upon arrival and various other entrance screening procedures [164] are also largely ineffective. Such border controls are akin to confiscating matches after the forest is already ablaze. Experience, including lessons learned during this pandemic, suggests that imposition of travel restrictions also generally fails to prevent the spread of new genetic variants, as their discovery typically lags well behind their emergence, and local detection often depends more on which locations are conducting routine genomic surveillance than on where the new variants actually originate [131].

### 8: ﻿Reassess testing practices and policies

Experience suggests that choice of diagnostic technologies should be determined by the intended use, whether to detect infection in individuals with suspected clinical symptoms or to identify potentially infectious individuals to inform isolation recommendations and conduct contact tracing. RT-PCR-based assays have so far been the preferred method for most such purposes [170]. Rapid antigen tests, which are both cheaper and faster, can lead to false negatives, especially in pre-symptomatic carriers, and when conducted without adequate quality control procedures. However, if performed correctly in appropriate populations, they may be sufficiently sensitive and specific for detecting potential infectivity [171], thus suggesting that antigen tests should increasingly be utilized for public health screening. Moreover, further investigation is needed regarding the extent to which positive SARS-CoV-2 RT-PCR results do not always reflect actual infectiousness [172–174], particularly among vaccinated or asymptomatic persons. Finally, given that vaccination reduces symptomatic and asymptomatic SARS-CoV-2 infections and that vaccinated individuals are likely to be less infectious if infected [175–177], testing and quarantine of vaccinated (or previously infected) persons following exposure to someone with suspected or confirmed COVID-19 should in general only be needed if COVID-19 symptoms develop [178]. As we increasingly recognize that SARS-CoV-2 is gradually becoming an endemic virus, it is vital to deemphasize identification of new cases as the key outcome metric of mitigation measures and rather to assess mortality and hospitalization rates [179]. This is also relevant considering that the vaccines were developed to reduce severe and fatal outcomes from COVID-19 and not to fully prevent onward transmission and infection.

### 9: ﻿Expand access to outpatient therapies and prophylactics

As with vaccines, the pandemic has presented challenges in identifying effective therapeutics on a greatly accelerated timeline. Although vaccination remains the priority, some vaccinated individuals will still contract SARS-CoV-2, and some persons will remain unvaccinated. While some medications have been tentatively permitted (not without controversy) on a compassionate use basis in a few countries, approved outpatient therapies for COVID-19 have been limited in most places to intravenous monoclonal antibodies, which are cost-prohibitive in most settings globally and often pose other considerable challenges for widespread use. As evidence on treatment options evolves, policymakers should prioritize quick access to effective outpatient therapies in patients with risk factors for severe disease and to prophylactics for unvaccinated persons at high risk. Assessment of previously identified safe medications might be an efficient way to quickly identify new therapies [180]. In addition, more research is urgently needed regarding the prevalence, diagnosis, prognosis, and treatment options for longer-term (“long haul”) COVID-19 complications.

### 10: ﻿Prevent and prepare for future pandemics

COVID-19 is the second major respiratory viral pandemic in just over a decade and the third coronavirus pandemic within 2 decades. More pandemics are likely in the coming years, whether from new coronaviruses and/or from other pathogens. We clearly must do everything possible to prevent and be better prepared for future pandemics and other public health emergencies [181, 182], and must learn and apply lessons from the recent experience with mitigating COVID-19.

Regarding prevention, policymakers need to take prudent actions immediately to reduce the likelihood of future pandemics, including addressing environmental destruction that brings different species into closer contact with humans, restricting the trafficking of animals, and strengthening biosecurity in laboratories that work with potential human pathogens.

Preparation for the next pandemics should include detailed plans by international organizations that are widely vetted and agreed upon. Lockdowns and quarantines, when (and only if) necessary, need to be designed equitably and to include protection, prioritization, and compensation for those most vulnerable [7], including the elderly, the poor, and workers in frontline and informal jobs. Effective mechanisms must also be established to address equity in access to treatments and vaccines, prioritizing those at highest risk. We certainly must avoid another situation where public health authorities and politicians are left to fly blind and then try to clean up the damage later. It would be a grave error to respond to a new pandemic without applying lessons from the current one.

## Conclusions

Given the high transmissibility of SARS-CoV-2, its continuing widespread circulation in some regions, and the emergence of new viral variants [147], it is unlikely that SARS-CoV-2 will be eradicated. Therefore, we will need to continue focusing on mitigation strategies, particularly vaccination [131]. Although SARS-CoV-2 genetic variants will keep emerging, vaccines have so far largely retained their ability to prevent fatal and other severe COVID-19 outcomes [183, 184]. Concerns that such variants will soon evade current vaccines may be overstated, as both the mRNA and adenovirus-DNA vaccines encode for the entire spike protein, providing robust and complex antibody-mediated as well as T-cell immune responses [17, 21, 185, 186]. Furthermore, vaccines can be rapidly modified, if necessary, to adapt to future variants [183, 184]. As previously noted, it is crucial to focus on the key public health objectives of preventing death and other severe disease outcomes, rather than continuing to use numbers of reported cases as the main metric. In any event, maintaining a constant state of emergency until the pandemic is over is not viable. Public health decision-making requires transparency and debate, which are often precluded by emergency orders. A more realistic public health goal is to adjust mitigation and treatment goals as the pandemic evolves, minimizing negative outcomes including the unintended harms associated with unfocused or irrelevant control efforts. The foregoing suggestions for refining our current approaches are presented as best practices that will nevertheless require continuous adjustment through reassessment of the latest evidence. We offer these in the reasonable hope of widespread vaccination helping to achieve far greater control of COVID-19, and also that the world will be better prepared for the next pandemic.

## Data Availability

Not applicable.

## Abbreviations

CDC: US Centers for Disease Control and Prevention
COVID-19: Coronavirus disease 2019
HCW: Healthcare worker
RCT: Randomized controlled trial
RT-PCR: Reverse transcriptase-polymerase chain reaction
SARS: Severe acute respiratory syndrome
SARS-CoV-2: Severe acute respiratory syndrome coronavirus 2
UNESCO: United Nations Educational, Scientific and Cultural Organization
UNICEF: United Nations Children’s Fund
WHO: World Health Organization
